# Factors Associated With 90‐Day Functional Outcome After Neurosurgical Management of Hypertensive Intracerebral Hemorrhage

**DOI:** 10.1002/brb3.71794

**Published:** 2026-09-23

**Authors:** Ali Kamal Sulaiman, Arshad Ali, Ali Msheik, Ibrahim Abdelhafiz, Fatima Al‐Sada, Maha Hamoud, Ghaya Al‐Rumaihi

**Affiliations:** ^1^ Department of Neurosurgery, Neuroscience Institute Hamad Medical Corporation Doha Qatar; ^2^ Department of Clinical Academic Sciences, College of Medicine Qatar University Doha Qatar; ^3^ Department of Neurological Sciences Weill Cornell Medicine Doha Qatar; ^4^ Department of Otolaryngology–Head and Neck Surgery National Golan Hospital Qunaiterah Syria

**Keywords:** functional outcome, Glasgow Coma Scale, hypertensive intracerebral hemorrhage, modified Rankin Scale, neurosurgical management, ordinal logistic regression

## Abstract

**Background:**

Prognostic factors after intracerebral hemorrhage (ICH) are well described, but evidence from surgically selected hypertensive ICH cohorts remains limited. This study examined preoperative clinical, hemodynamic, and radiographic factors associated with the full 90‐day modified Rankin Scale (mRS) distribution in patients who underwent neurosurgical management for hypertensive ICH.

**Methods:**

This retrospective single‐center cohort used a prospectively maintained stroke registry at Hamad Medical Corporation, Qatar, from 2013 through 2024. The registry contained 113 adults who underwent neurosurgical management for hypertensive ICH. The primary outcome was 90‐day mRS (0–6). Because mRS is ordinal, the primary analysis used cumulative‐logit ordinal logistic regression. The prespecified model included age, admission mean arterial pressure (MAP), admission pulse pressure, admission Glasgow Coma Scale (GCS) score, first preoperative CT hematoma volume, and midline shift. Complete‐case analysis was used.

**Results:**

Ninety‐day mRS was available for 95 of 113 patients; 87 had complete data for the primary model. Median 90‐day mRS was 4 [IQR, 3–5]. Older age was associated with greater odds of a worse 90‐day mRS category (adjusted OR per 10 years, 1.58; 95% CI, 1.10–2.27; *p* = 0.012), whereas higher admission GCS was associated with lower odds of a worse category (adjusted OR per point, 0.88; 95% CI, 0.79–0.97; *p* = 0.013). The confidence intervals for MAP, pulse pressure, hematoma volume, and midline shift included the null.

**Conclusions:**

In this surgically selected hypertensive ICH cohort, older age and lower admission GCS were associated with worse 90‐day functional status. The study does not establish new prognostic factors, treatment benefit, or a validated prediction model; rather, it provides cohort‐specific evidence using an analysis aligned with the ordinal structure of mRS.

## Graphical abstract

In this surgically selected cohort of hypertensive intracerebral hemorrhage, older age and lower admission Glasgow Coma Scale scores were independently associated with worse 90‐day functional status on ordinal mRS analysis. These findings support established prognostic relationships but do not represent a validated prediction model or an estimate of surgical benefit.

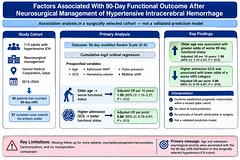

## 1 Introduction

Intracerebral hemorrhage (ICH) remains a major cause of death and long‐term disability. Contemporary guidelines emphasize that outcome is shaped by baseline neurological severity, hematoma characteristics, intraventricular extension, physiological instability, treatment course, and complications (Greenberg et al. 2022; Steiner et al. 2025). Prognostic instruments such as the ICH score and Functional Outcome in Patients with Primary Intracerebral Hemorrhage (FUNC) score demonstrate the importance of age and neurological status, but these tools were not developed specifically to characterize outcomes in a surgically selected hypertensive ICH population (Hemphill et al. 2001; Rost et al. 2008).

Hypertension is a major cause of spontaneous ICH, particularly in deep brain structures. Admission blood‐pressure burden, hematoma volume, and mass effect are biologically plausible markers of early injury severity, while admission Glasgow Coma Scale (GCS) provides a bedside summary of neurological compromise (Greenberg et al. 2022; Davis et al. 2006; Anderson et al. 2013; Qureshi et al. 2016; Manning et al. 2014). Surgical cohorts are additionally affected by treatment‐selection mechanisms: patients are selected for intervention on the basis of clinical severity, hemorrhage anatomy, mass effect, ventricular involvement, and local practice. Consequently, associations observed after surgery should not be interpreted as estimates of surgical benefit or as evidence that one operative strategy is superior (Steiner et al. 2025; Mendelow et al. 2005).

Previous studies have already established many prognostic correlates of ICH outcome; therefore, the purpose of this study is not to claim the discovery of novel predictors. Its contribution is narrower: to describe the 90‐day functional‐outcome distribution in a relatively young, surgically selected hypertensive ICH cohort from Qatar and to examine whether a predefined set of routinely available preoperative variables is associated with the full ordinal mRS distribution using a statistically appropriate ordinal model. We hypothesized that greater baseline injury severity would be associated with worse 90‐day functional status.

## 2 Methods

### 2.1 Study Design and Setting

This retrospective single‐center cohort study was conducted at Hamad Medical Corporation (HMC), Doha, Qatar, using a prospectively maintained institutional stroke registry covering January 2013 through December 2024. HMC is the national tertiary referral system providing comprehensive stroke and neurosurgical care.

### 2.2 Eligibility and Cohort Definition

Adults (≥18 years) with hypertensive ICH who underwent neurosurgical management were eligible. The source registry and electronic medical record were used to exclude secondary causes of hemorrhage, including vascular malformation, aneurysm, tumor, coagulopathy‐related secondary hemorrhage, and trauma, as described in the original cohort definition. The available dataset contained 113 eligible patients. The cohort was restricted to treated patients and did not include a nonoperative comparison group; accordingly, all analyses address associations with outcome within the treated cohort rather than the effectiveness of surgery.

### 2.3 Outcome Ascertainment

The primary outcome was functional status at 90 days measured with the modified Rankin Scale (mRS), an ordered seven‐category scale from 0 (no symptoms) to 6 (death). Registry values coded as death were represented as mRS 6 for analysis. Ninety‐day mRS was available for 95 of 113 patients. Outcome information in the source cohort was obtained from documented follow‐up or structured post‐stroke follow‐up records.

### 2.4 Candidate Variables

Six variables were retained in the primary model because they were available in a sufficiently harmonized form across the study period and were clinically relevant before or at the time of neurosurgical intervention: age, admission mean arterial pressure (MAP), admission pulse pressure, admission GCS, first preoperative Computed Tomography (CT) hematoma volume, and midline shift. Hematoma volume was measured from CT using the conventional ABC/2 approach in the source dataset. Continuous variables were kept continuous in the primary model to avoid information loss from arbitrary dichotomization.

### 2.5 Operative, Radiographic, and Medical‐Management Data Availability

The registry contained additional operative and radiographic fields, but detailed procedure subtype, surgical timing, ventricular‐cast burden, postoperative hematoma volume, hematoma‐clearance percentage, and components of concomitant medical management were not available in a sufficiently complete and harmonized form for reliable inclusion in the primary analysis. These variables were therefore not imputed or reconstructed. Their absence limits adjustment for treatment‐selection and perioperative factors and is explicitly considered when interpreting the findings.

### 2.6 Statistical Analysis

Descriptive statistics were reported according to the observed distribution of each variable. The primary inferential analysis was cumulative‐logit ordinal logistic regression with 90‐day mRS as the ordered outcome. Covariates were scaled as age per 10 years, admission MAP per 10 mmHg, admission pulse pressure per 10 mmHg, admission GCS per point, CT hematoma volume per 10 cm^3^, and midline shift per millimeter. An odds ratio (OR) greater than 1 indicates greater odds of being in a worse mRS category. Complete‐case analysis was used; 87 patients had complete outcome and covariate data for the primary model. No linear‐regression analysis of raw mRS and no multivariable binary prediction models are presented in this revision because those approaches either impose additional measurement assumptions or were unstable relative to the available sample size and outcome distribution. The archived reanalysis output available for this revision did not include a formal proportional‐odds diagnostic; therefore, the proportional‐odds assumption is acknowledged as an unverified model assumption rather than being claimed as tested. Two‐sided *p* < 0.05 was used for statistical inference, and emphasis was placed on effect estimates and 95% confidence intervals (CIs) rather than model‐based prediction.

### 2.7 Ethics

The Institutional Review Board at HMC approved the study with a waiver of informed consent because of its retrospective design and secondary use of de‐identified registry data.

## 3 Results

### 3.1 Participant Flow

The cohort contained 113 surgically managed patients. Ninety‐day mRS was available for 95 patients (84.1%). The primary ordinal regression included 87 complete cases (77.0% of the full cohort and 91.6% of those with a recorded 90‐day mRS).

### 3.2 Baseline and Outcome Characteristics

The cohort was relatively young, with a mean age of 46.8 ± 10.4 years. Admission GCS had a median of 9 [IQR, 6–14], and first preoperative CT hematoma volume had a median of 44.0 cm^3^ [IQR, 26.5–65.3]. Median 90‐day mRS among patients with available outcome data was 4 [IQR, 3–5]. Descriptive characteristics are shown in Table 1, and the 90‐day mRS distribution is shown in Table 2.

**TABLE 1:** Descriptive characteristics.

Variable	Available N	Mean ± SD	Median [IQR]	Range
Age (years)	113	46.80 ± 10.40	47.00 [39.00–53.00]	28.00–90.00
Admission SBP (mmHg)	113	193.88 ± 36.19	190.00 [170.00–215.00]	115.00–276.00
Admission DBP (mmHg)	110	115.37 ± 22.92	114.00 [100.00–127.75]	67.00–172.00
Admission MAP (mmHg)	110	141.65 ± 25.69	138.67 [125.08–157.58]	83.00–197.00
Admission pulse pressure (mmHg)	110	78.85 ± 24.71	77.00 [58.50–92.00]	30.00–161.00
Admission GCS	113	9.38 ± 4.13	9.00 [6.00–14.00]	3.00–15.00
CT1 hematoma volume (cm^3^)	107	49.61 ± 33.64	44.00 [26.50–65.25]	2.80–217.00
Midline shift (mm)	107	7.69 ± 4.16	8.00 [4.90–10.00]	0.00–23.00
90‐day mRS	95	:	4.00 [3.00–5.00]	0–6

**TABLE 2:** Distribution of 90‐day modified Rankin Scale scores.

mRS category	Valid‐outcome cohort, n (%)	Primary model cohort, n (%)
0	4 (4.2%)	3 (3.4%)
1	1 (1.1%)	1 (1.1%)
2	8 (8.4%)	8 (9.2%)
3	15 (15.8%)	14 (16.1%)
4	27 (28.4%)	25 (28.7%)
5	19 (20.0%)	18 (20.7%)
6	21 (22.1%)	18 (20.7%)

### 3.3 Primary Ordinal Analysis

The cumulative‐logit ordinal model included 87 complete cases. Older age was associated with greater odds of worse 90‐day functional status (adjusted OR per 10 years, 1.58; 95% CI, 1.10–2.27; *p* = 0.012). Higher admission GCS was associated with lower odds of a worse mRS category (adjusted OR per point, 0.88; 95% CI, 0.79–0.97; *p* = 0.013). The 95% CIs for admission MAP, pulse pressure, hematoma volume, and midline shift included 1.0 (Table 3).

**TABLE 3:** Primary cumulative‐logit ordinal regression for worse 90‐day mRS category.

Predictor and unit	Adjusted OR	95% CI	*p* value
Age (per 10 years)	1.58	1.10–2.27	0.012
Admission MAP (per 10 mmHg)	1.18	1.00–1.40	0.053
Admission pulse pressure (per 10 mmHg)	1.07	0.91–1.27	0.415
Admission GCS (per point)	0.88	0.79–0.97	0.013
CT1 hematoma volume (per 10 cm^3^)	1.13	0.99–1.30	0.077
Midline shift (per 1 mm)	1.08	0.97–1.21	0.152

*Note*: OR >1 indicates greater odds of being in a worse mRS category. The model used 87 complete cases.

## 4 Discussion

### 4.1 Key Findings

In this surgically selected hypertensive ICH cohort, the 90‐day disability burden was substantial: the median mRS was 4, and 42.1% of the patients with recorded outcomes were in mRS categories 5–6. In the primary ordinal model, older age and lower admission GCS were associated with worse 90‐day functional status. MAP, pulse pressure, hematoma volume, and midline shift had CIs that crossed the null after adjustment. These results should be interpreted as within‐cohort associations, not as a validated prediction model.

### 4.2 Interpretation and Comparison With Existing Literature

The association between lower GCS and worse functional outcome is consistent with established ICH prognostic frameworks. GCS is central to the ICH score and reflects the combined clinical consequences of primary tissue injury, mass effect, elevated intracranial pressure, and global neurological compromise (Hemphill et al. 2001; Gregório et al. 2021). Age is also an established determinant of post‐ICH recovery and is incorporated into functional prognostic tools such as the FUNC score (Rost et al. 2008). The present study therefore confirms, rather than newly discovers, the relevance of these factors in a treated hypertensive ICH cohort.

Hematoma volume is strongly associated with mortality and poor functional outcome in broader ICH populations (Davis et al. 2006; Cindea et al. 2025). In the present adjusted ordinal model, the estimated association for hematoma volume was in the expected direction but imprecise (OR per 10 cm^3^, 1.13; 95% CI, 0.99–1.30). This should not be interpreted as evidence that hematoma volume is unimportant. The surgically selected design, modest complete‐case sample, correlation among markers of injury severity, and absence of harmonized information on intraventricular extension, surgical timing, and postoperative residual volume may attenuate or destabilize individual estimates.

Admission blood pressure has a complex relationship with ICH outcome. Randomized trials of acute blood‐pressure reduction have not shown a simple one‐to‐one relationship between a single admission pressure measurement and long‐term recovery (Anderson et al. 2013; Qureshi et al. 2016). In this cohort, MAP showed a borderline association with worse ordinal outcome (OR per 10 mmHg, 1.18; 95% CI, 1.00–1.40; *p* = 0.053), while pulse pressure was not clearly associated after adjustment. These estimates are best viewed as descriptive of the cohort rather than evidence for a treatment threshold.

### 4.3 Clinical Implications

The practical implication is modest but clinically relevant: age and admission GCS remain useful descriptors of baseline prognosis even among patients selected for neurosurgical treatment. The results do not provide a rule for deciding who should undergo surgery, do not quantify benefit attributable to surgery, and do not justify choosing one operative technique over another. They also should not be used in isolation for treatment limitation. Prognostication after ICH requires the integration of neurological examination, hemorrhage anatomy, ventricular involvement, physiological course, comorbidity, treatment response, and rehabilitation potential (Greenberg et al. 2022; Steiner et al. 2025; Keep et al. 2012).

### 4.4 Future Perspective

Future work should use a prospectively defined surgical ICH dataset with standardized recording of hematoma location, intraventricular hemorrhage and ventricular‐cast burden, operative indication and timing, exact procedure subtype, preoperative and postoperative hematoma volumes, complications, critical‐care therapies, and rehabilitation exposure. A larger multicenter sample would permit prespecified validation of ordinal models, assessment of the proportional‐odds assumption, nonlinear effects and interactions, and internal or external validation if a genuine prediction model is developed. The current dataset is better suited to association analysis than to model development.

### 4.5 Strengths and Limitations

Strengths include use of patient‐level registry data, a clinically defined treated cohort, preservation of continuous predictors in the primary analysis, and use of an ordinal model aligned with the measurement structure of mRS. The revised analysis also avoids presenting an in‐sample Area under the curve (AUC) or linear mRS model as evidence of predictive performance.

The study has important limitations. It is retrospective, single‐center, and restricted to patients selected for neurosurgical treatment, which limits generalizability and introduces treatment‐selection bias. Eighteen of 113 patients lacked a recorded 90‐day mRS, and the primary model used 87 complete cases; outcome and covariate missingness may therefore introduce bias. The cohort is younger than many international ICH populations. Important covariates including detailed hematoma location, intraventricular/ventricular‐cast burden, surgical timing, exact procedure subtype, postoperative hematoma volume and clearance, standardized medical‐management exposures, complications, and rehabilitation intensity were not harmonized sufficiently for reliable multivariable adjustment. The sample is also modest, particularly in the least‐disabled mRS categories. Finally, the archived ordinal‐regression output did not contain a formal proportional‐odds diagnostic, so assumption could not be verified in this revision. These limitations preclude claims of a validated prediction model or causal inference.

## 5 Conclusion

Among patients who underwent neurosurgical management for hypertensive ICH, older age and lower admission GCS were associated with worse 90‐day functional status on ordinal mRS analysis. These findings confirm established prognostic relationships within a surgically selected Qatar cohort rather than identify novel predictors. The results are hypothesis‐generating and should be interpreted in light of missing follow‐up, incomplete perioperative covariate harmonization, and the absence of a nonoperative comparator or external validation.

## Author Contributions

**Arshad Ali**: conceptualization, investigation, Writing – original draft, Writing – review and editing. **Ibrahim Abdelhafiz**: conceptualization, investigation, project administration, formal analysis, data curation, writing – review and editing. **Ghaya Al‐rumaihi**: conceptualization, investigation, writing – original draft, writing – review and editing, visualization, formal analysis, project administration, resources, data curation, software, supervision, methodology, validation. **Maha Hamoud**: conceptualization, writing – original draft, project administration, formal analysis, software, data curation, methodology, validation, writing – review and editing. **Ali Msheik**: investigation, conceptualization, visualization, writing – review and editing, writing – original draft, validation, software, data curation, methodology, resources, project administration. **Ali Kamal Sulaiman**: conceptualization, investigation, funding acquisition, writing – original draft, resources, validation, writing – review and editing, methodology. **Fatima Al‐Sada**: investigation, conceptualization, project administration, formal analysis, validation, methodology, data curation, resources, writing – review and editing, writing – original draft. All authors contributed substantially to the work, critically reviewed the manuscript for important intellectual content, approved the final version of the manuscript, and agreed to be accountable for all aspects of the work.

## Funding

The authors have nothing to report.

## Conflicts of Interest

The authors declare no conflicts of interest.

## Data Availability

Data are available upon justified request.
